# Applied systems thinking: a viable approach to identify leverage points for accelerating progress towards ending neglected tropical diseases

**DOI:** 10.1186/s12961-020-00570-4

**Published:** 2020-06-03

**Authors:** Jeffrey Glenn, Kimberly Kamara, Zaiyanatu Abubakar Umar, Teresa Chahine, Nils Daulaire, Thomas Bossert

**Affiliations:** 1 Department of Public Health, College of Life Sciences, Brigham Young University, 2032 LSB, Provo, UT United States of America; 2 The END Fund, New York, NY United States of America; 3 Neglected Tropical Diseases Division, Federal Ministry of Health, Abuja, Nigeria; 4 Yale School of Management, New Haven, CT United States of America; 5 Harvard T.H. Chan School of Public Health, Boston, MA United States of America

**Keywords:** systems thinking, systems change, neglected tropical diseases, global health, public health, causal loop diagrams

## Abstract

**Background:**

Systems thinking is a conceptual approach that can assist stakeholders in understanding complexity and making progress on persistent public health challenges. Neglected tropical diseases (NTDs), a complex global health problem, are responsible for a large disease burden among impoverished populations around the world. This aim of this study was to better discern the many complexities of the global NTD system in order to identify and act on leverage points to catalyse progress towards ending NTDs.

**Methods:**

Existing frameworks for systems change were adapted to form the conceptual framework for the study. Using a semi-structured interview guide, key informant interviews were conducted with NTD stakeholders at the global level and at the country level in Nigeria. The interview data were coded and analysed to create causal loop diagrams that resulted in a qualitative model of the global NTD system.

**Results:**

The complete qualitative model is discussed and presented visually as six separate sub-components that highlight key forces and feedback loops within the global NTD system.

**Conclusions:**

We identified five leverage points for NTD system change, namely (1) clarify the potential for and assess realistic progress towards NTD elimination, (2) increase support for interventions besides drug delivery, (3) reduce dependency on international donors, (4) create a less insular culture within the global NTD community, and (5) systemically address the issue of health worker incentives. The specific findings for NTDs raise a number of uncomfortable questions that have not been addressed, at least in part, because it is easier to continue focusing on ‘quick win’ solutions. The study provides a model of a systems thinking approach that can be applied to other complex global health and development challenges in order to understand complexity and identify leverage points for system change.

## Background

Neglected tropical diseases (NTDs) are an example of a global health issue for which sustainable progress will depend on stakeholders’ ability to think systemically about potential solutions. Along with many of the world’s most persistent public health challenges, NTDs can be considered as ‘complex’, ‘wicked’ or ‘adaptive’ problems [1, 2]. These types of problems and their root causes are often difficult to define and their solutions are typically unclear [3]. Rather than relying on blueprint approaches or technical solutions, making headway on complex problems requires a whole systems perspective that incorporates adaptation and group learning [4–7]. Systems thinking is a conceptual approach that seeks a better understanding of complexity in order to increase the ability of stakeholders to influence durable change to address complex problems [8–11].

### Systems thinking

At its core, a system is a collection of parts that, through their interactions, form a whole with properties beyond the sum of the component parts [12, 13]. Natural and human systems have high levels of dynamic complexity that arises from the interaction of multiple agents over time [14]. Many health sector problems are considered complex due to the interconnectedness of highly heterogenous groups of actors operating with multiple interrelated and simultaneous strategies in a constantly changing context [7, 15, 16]. Gaining a holistic perspective of complex problems embedded in complex systems is critical to identifying solutions with the potential for lasting change [4, 6, 10]. Systems thinking is a discipline for expanding our understanding of complex situations by seeing wholes, patterns and interrelationships rather than separate system parts [6]. Thinking in this way allows us to identify root causes of problems and see new opportunities for progress [17]. In approaching problems and solutions through this lens, systems thinking helps us to overcome the linear and reductionist approaches commonly applied to problem solving in the social sectors and to reinvent our policies and institutions according to this holistic, dynamic view [5].

In practice, systems thinking includes a broad array of qualitative and quantitative methods and tools designed to better understand system behaviours and intervene in the context of complexity and uncertainty [18]. Many systems thinking tools are designed to assist stakeholders in co-producing knowledge about a system and in visually capturing and communicating this information [19–21]. On the qualitative side, soft systems methodology uses the idea of complexity as an interrogative tool for stimulating debate and learning, building relationships, and galvanising people into action [19, 22, 23]. The focus with this approach is on engaging multiple stakeholders in developing ‘rich pictures’ of a problem situation that help to make explicit the mental models held by those with differing perspectives [5, 7, 20, 22]. On the quantitative side, system dynamics modelling is a methodologically demanding tool that incorporates data into simulations of system behaviour in order to describe a system and its operations [14, 20]. These models allow testing of hypotheses in the context of complexity in order to help identify the few key areas in which policy-makers should focus their attention [5, 24]. System dynamic modelling also allows users to see how the system may evolve and how changes may affect it over time [25]. System dynamics models built through group processes are useful in harnessing the perspectives and insights of stakeholders with the most experience interacting with the systems addressed in the models [24].

Causal loop diagrams are essential components of quantitative system dynamic models, but they can also be used to build stand-alone qualitative system models that visually map feedback loops depicting the interactions of actors, linkages and relationships that characterise the whole system [26, 27]. Although qualitative models lack the capacity for quantitative simulations that represent how systems change over time, they are a useful ‘snapshot’ of systems that can aid in understanding complex problems and exploring potential leverage points for intervention and for systems change [4, 25, 28]. These models serve as a starting point for engagement between multiple stakeholders to sift out major issues, promote inquiry and challenge preconceived ideas [15, 29, 30]. Some have suggested that the power of qualitative modelling lies in encouraging decision-makers to avoid blind spots and to take action [31].

Although systems change is a term often used to refer generally to different types of big picture initiatives intended to solve social problems, some scholars have offered more precise definitions [4, 19, 20, 32, 33]. Specifically, Foster-Fishman et al. submit that systems change “*refers to an intentional process designed to alter the status quo by shifting and realigning the form and function of a targeted system*” [19]. Various theoretical frameworks exist for understanding complexity and working towards systems change [4, 15, 19]. Foster-Fishman et al. [19] use insights from their work in community development to outline detailed steps for defining and assessing the systemic nature of a problem. These steps culminate into a process for identifying feasible levers for change within the patterns that have been uncovered. Rwashana et al.’s [15] revised dynamic synthesis methodology begins with a qualitative research approach – using interviews and surveys – to gain a deeper understanding of the complex problem of interest in order to then start working towards systems change.

Systems thinking is gaining increased attention in the field of public health, where interventions and policies often fail to adequately take into account features of dynamic complexity, resulting from a combination of the diseases or conditions themselves and the systems in which they are embedded, that make public health challenges so difficult to solve [7, 15, 26, 34]. Because key challenges in public health are fundamentally systems problems, it is common for well-intended interventions to have counterintuitive and unintended negative consequences [18, 35]. In the public health context, systems have been defined as the interconnected sets of actors, activities and settings that jointly produce a health outcome [19, 25]. In 2009, WHO published a report making the case that, due to the complexity inherent to health systems, “*every intervention, from the simplest to the most complex, has an effect on the overall system, and the overall system has an effect on every intervention*” [27]. Thus, this report and subsequent publications argue that systems thinking has enormous potential to help decipher health system complexity and to design effective responses that seek to account in a holistic manner for the unpredictability of operating within this context [5, 26, 34, 36, 37]. While not a panacea, systems thinking tools can be useful in addressing complex public health problems by identifying key blockages and challenges and in helping to consider the long-term consequences of our actions.

Various systems thinking approaches have been applied to complex public health issues. Researchers have developed quantitative system dynamics models to improve planning for chronic disease-related interventions focused on diabetes, cardiovascular disease and tobacco control [8, 18, 38, 39]. Similar models have been applied to understand the complexities of eradicating infectious diseases such as polio and, more recently, of stopping the Ebola virus outbreak in West Africa [40, 41]. Without incorporating quantitative simulations, others have applied qualitative tools (i.e. causal loop diagramming) to illustrate the underlying dynamics of a variety of disease- or condition-specific interventions, such as improving newborn health or increasing vaccination rates, as well as health system strengthening interventions such as efforts to build resilient health systems, implement innovative financing mechanisms and assess programme sustainability [15, 28, 31, 42–44]. Despite the growing interest in systems thinking and the potential it holds for improving public health, there remains a need for additional documented, practical attempts at utilising systems thinking approaches to address real-world public health problems in ways that bring together diverse perspectives to influence policy and practice [19, 35]. A recent review found that a large proportion of public health articles drawing on systems methodologies were commentaries or calls for the application of these methods to public health [36]. The same review suggests that qualitative modelling techniques, such as the approach we apply in this study, are likely to be the most useful addition to public health.

### Neglected tropical diseases

As a complex public health problem for which the global health community often focuses on a narrow set of interventions, NTDs represent an ideal case study for the practical application of systems thinking. NTDs encompass 20 bacterial and parasitic infections that continue to represent a major disease burden in many parts of the world [45]. These diseases are closely associated with poverty, with the majority of the burden in sub-Saharan Africa and among the poor in middle-income countries [46, 47]. It is estimated that, annually, these diseases cause approximately 350,000 deaths and are responsible for 27 million disability-adjusted life years lost [48]. The focus of global NTD control and elimination efforts has largely been on the five NTDs that can be prevented through preventive chemotherapy medicines distributed via mass drug administration (MDA), namely lymphatic filariasis, onchocerciasis, soil-transmitted helminths, schistosomiasis and trachoma [49]. NTDs have generated significant attention from aid agencies and philanthropists in part because NTD control and elimination is widely seen as one of the best buys in global health, with effective MDA interventions in many circumstances costing less than US$0.50 per person per year [48, 49].

Although NTDs are often portrayed as a simple problem with known and easily implementable solutions (i.e. preventive chemotherapy drugs), many complex challenges have yet to be fully understood and addressed in order for countries to reach the ambitious 2020 NTD ‘end goals’ that were agreed upon by the World Health Assembly in 2013 [48]. Achieving these goals will require coordination between multiple actors engaged in numerous interrelated projects, which include changing attitudes, perceptions and practices of multiple stakeholders [15]. For example, despite significant financial and in-kind contributions, there is still an estimated US$200 million gap in annual funding needed to reach global NTD targets [50]. The level of political priority accorded to NTDs remains relatively low, particularly at the country level, compared to many other global health issues [51]. High-burden countries often lack the capacity to manage complicated supply chains and deliver available treatments, which creates a situation where the availability of donated drugs can outstrip country capacity to deliver them [52, 53]. Comorbidities exist with other diseases that make MDA interventions harder to implement in certain geographic areas [54]. Additionally, many NTD activities continue to be carried out in siloes rather than being integrated within country health systems and with donor programmes for other diseases and development priorities (e.g. water and sanitation) [55]. These represent a few of the various reasons why NTDs are a complex problem that will require learning on behalf of all stakeholders to generate additional, innovative solutions.

Our study seeks to encourage the use of systems thinking in public health by demonstrating how a systems thinking approach – in the form of qualitative modelling – can be applied to global NTD control and elimination efforts. In addition to focusing at the global level, our study looks at Nigeria as an example country. Nigeria has the highest burden of NTDs among African countries, with over 130 million people requiring preventive chemotherapy treatment annually [56]. Nigeria also receives a large amount of donor funding for NTDs, leading to significant programmatic complexity due to large numbers of domestic and international organisations involved in NTD control and elimination efforts in the country [57–59]. The overall aim of this study is to better discern the complexity of the global NTD system in order to identify and act on leverage points – “*places within a complex system where a small shift in one thing can produce big changes in everything*” [60] – to catalyse progress towards ending NTDs. Drawing on systems theory, the specific research questions that drive the study and frame our approach are (1) in which ways, if any, are NTDs a systemic or complex problem? (2) What leverage points for systems change exist within the global movement to end NTDs?

## Methods

### Conceptual framework

To address the study objectives, we developed a conceptual framework for systems change (Table 1) based on systems thinking theory and drawing heavily on Foster-Fishman et al.’s [19] framework as well as elements of the dynamic synthesis methodology framework used by Rwashana et al. [15].

**Table 1:** Conceptual framework for systems change

Project phases	Project activities
Phase 1: Refine conceptual framework	- Literature review - Informal discussions with global NTD organisations
Phase 2: Bound the system	- Desk review of NTD programme documents - Systems change workshops with NTD stakeholders
Phase 3: Assess dynamic interactions	- Key informant interviews - Qualitative data analysis - Qualitative model building
Phase 4: Identify levers for change	- Analysis of qualitative model - Model validation with NTD stakeholders
Phase 5: Mobilise for systems change	- Academic and non-academic publications - Presentations to NTD organisations

*NTD* neglected tropical diseases

Our project framework for systems change (Table 1) includes five phases, beginning with the development of the theoretical foundations of the project and then developing a logical justification for how the project could lead to change in addressing the problem of NTDs. Phase 1 began with a broad review of NTD and complex systems literature as well as discussions with NTD experts. Phase 2 consisted of a desk review of NTD programme materials gathered during the first phase as well as interactive workshops with global NTD organisations in order to define the NTD system and develop the research protocol. Phase 3 included qualitative data collection from key informant interviews with a diverse range of stakeholders. We then analysed these data, as described below, and used them to build a qualitative model of causal loop diagrams mapping out key variables and interactions within the global NTD system. In Phase 4, we applied this model to identify leverage points for intervention and systems change and we invited some stakeholders to provide feedback on the model and findings. Phase 5 is ongoing as we seek to disseminate the study results and encourage the NTD community to incorporate the findings into policy and practice. This final phase includes additional opportunities for stakeholders to provide feedback on the model as it serves as a launching point for promoting inquiry and learning.

### Participants

This qualitative study design relied on key informant interviews (*n* = 45) with a wide range of individuals holding key positions in global-level stakeholder organisations focused on addressing NTDs. To identify study participants, we first compiled a list of NTD stakeholder organisations. These organisations included bilateral and multilateral organisations, private sector companies, non-governmental organisations (NGOs), and research institutions. We purposively identified study participants at the global level from this pool of organisations based on two main considerations, namely selecting individuals (1) with experience and knowledge of the NTD sector and (2) from diverse types of organisations, who were likely to have unique perspectives.

We also conducted key informant interviews in Nigeria at national, state and community levels. The purposive sampling process for identifying participants was similar to the process we used at the global level. After reviewing programme documents and holding initial discussions with individuals familiar with the NTD programmes in Nigeria, we compiled a list of organisations and people to interview. This included representatives from federal, state and local governments, multilateral organisations, international and local NGOs, and community health facilities. We selected one Nigerian state, two local government areas within that state and one community within each of the two local government areas in which to conduct our interviews.

### Data collection

The majority of participants were contacted via email and interviewed via Skype. State and local participants were contacted vis-à-vis local NTD organisations and interviewed in person, with the exception of a few interviews conducted via telephone or skype. All participants received background information on the study and their rights as participants and then participants gave oral consent to proceed according to the research protocols reviewed and approved by the Institutional Review Board at the Harvard TH Chan School of Public Health and by the National Health Ethics Research Committee within the Federal Ministry of Health in Nigeria.

The lead author used a semi-structured interview guide to conduct in-depth key informant interviews with the research participants between December 2017 and February 2018. Consistent with the theory behind systems thinking, the interview guide sought to elicit perspectives from the various stakeholders about feedback loops and interactions between critical variables within the global NTD system. The guide included questions about what the respondents saw as the recent successes and challenges in addressing NTDs, examples of programmatic failures and potential changes that would have a positive impact on the overall system. Although all interviewees spoke English, at the community level, we were accompanied by translators fluent in local dialects, who clarified meanings of questions and responses when necessary. Interviews were audio recorded and then transcribed verbatim.

### Analysis

Interview transcripts were imported into MAXQDA, which we used to help organise and support the research-driven coding process. The research team developed a preliminary codebook based on the initial literature review and the project conceptual framework. The codebook included codes for variables cited as key forces in the system as well as codes for illustrating the interactions between variables. A single researcher then analysed and coded all interviews using the preliminary codebook, with emergent themes, variables and interactions being identified and added to the codebook throughout the analysis process. The same researcher then analysed and coded all interviews a second time using the final codebook. The variables and themes were discussed and modified regularly with the full research team throughout the coding and analysis process.

The authors used the coded interview data to identify the variables and relationships between variables that were reported as important factors driving and impeding progress towards NTDs. Using the coded data as a guide, the authors iteratively built causal loop diagrams for each of the central issues reported by respondents, specifically identifying reinforcing and balancing feedback loops. Variables in the model were connected only if that connection was explicitly made by the interview participants or backed by previous research. After building and refining a number of loops, the authors selected those that portrayed systemic patterns that were counterintuitive or that highlighted cases of unintended consequences for inclusion in the final model. These loops were combined into a single qualitative model that was built and visualised using Kumu, an online system mapping and visualisation platform. The authors then sought feedback on the model from stakeholders, including some key informant interview participants and multiple staff members at a major NTD NGO, and made adjustments based on stakeholder input.

## Results

Table 2 lists the number of interviews (45 total) conducted for each type of organisation at the global level (17 interviews) and at the country level (28 interviews).

**Table 2:** Interview respondents

Type of organisation	Number of interviews (***n*** = 45)
Global	*n* = 17
Non-governmental organisation funders and implementers	9
Multilateral and bilateral organisations	4
Pharmaceutical companies	3
Research institution	1
Nigeria	*n* = 28
Non-governmental organisation funders and implementers	8
Multilateral and bilateral organisations	2
Federal Ministry of Health	4
State government	2
Local government area	6
Local health and education facilities	3
Community drug distributors	2
Research institution	1

### Qualitative model

**Fig. 1:**
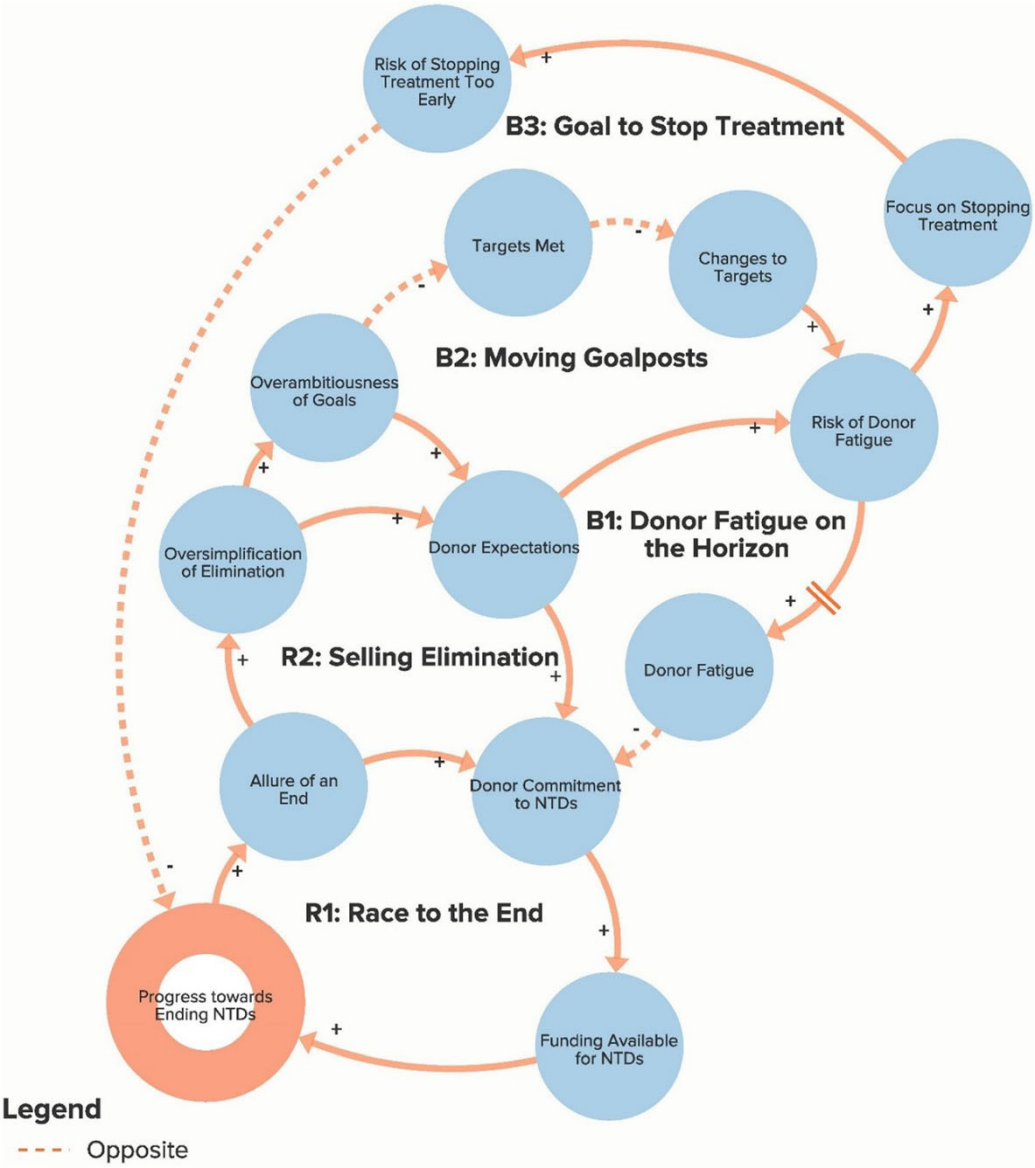
Sub-component 1: Global neglected tropical disease advocacy

The authors used data from the key informant interviews to create a qualitative model of the NTD system. The purpose of the qualitative model, or system map, is to illustrate key elements of the NTD system as reported by interview respondents in order to identify potential leverage points for intervention and change. Progress towards ending NTDs is the central goal of the system illustrated in the model. Each loop is labelled with a name that highlights the theme demonstrated by the loop. In our model, reinforcing loops (labelled with an R) are positive forces and lead to progress, while balancing loops (labelled with a B) are negative forces and stagnate progress. Because not all of our loops are fully self-correcting or self-reinforcing, our use of the terms reinforcing loops and balancing loops is less precise than definitions employed by systems theorists [14, 17]. The arrows in our model illustrate the direction of the causal link between variables. Variables linked with a plus (+) sign move in the same direction (i.e. more donor commitment leads to more funding for NTDs), while the variables linked with a minus (−) sign move in opposite directions (i.e. more drugs delivered leads to fewer cases of NTDs). The complete model is divided into six separate sub-components to highlight the following themes: Global NTD advocacy, Focus of NTD interventions, Political priority of NTDs, Global collaboration between NTD partner organisations, Community participation in NTD programmes, and Introducing systems change. To simplify presentation of the findings, the sub-components displayed in Figs. 1. 2, 3, 4, 5 and 6 show segments of the feedback loops rather than the isolated, closed feedback loops. The complete qualitative model that combines all six sub-components with closed loops and depicts the identified leverage points is shown at the end of this section in Fig. 7. The remainder of this section describes key insights from the model by summarising the positive and negative forces in each of the six sub-components.

**Fig. 2:**
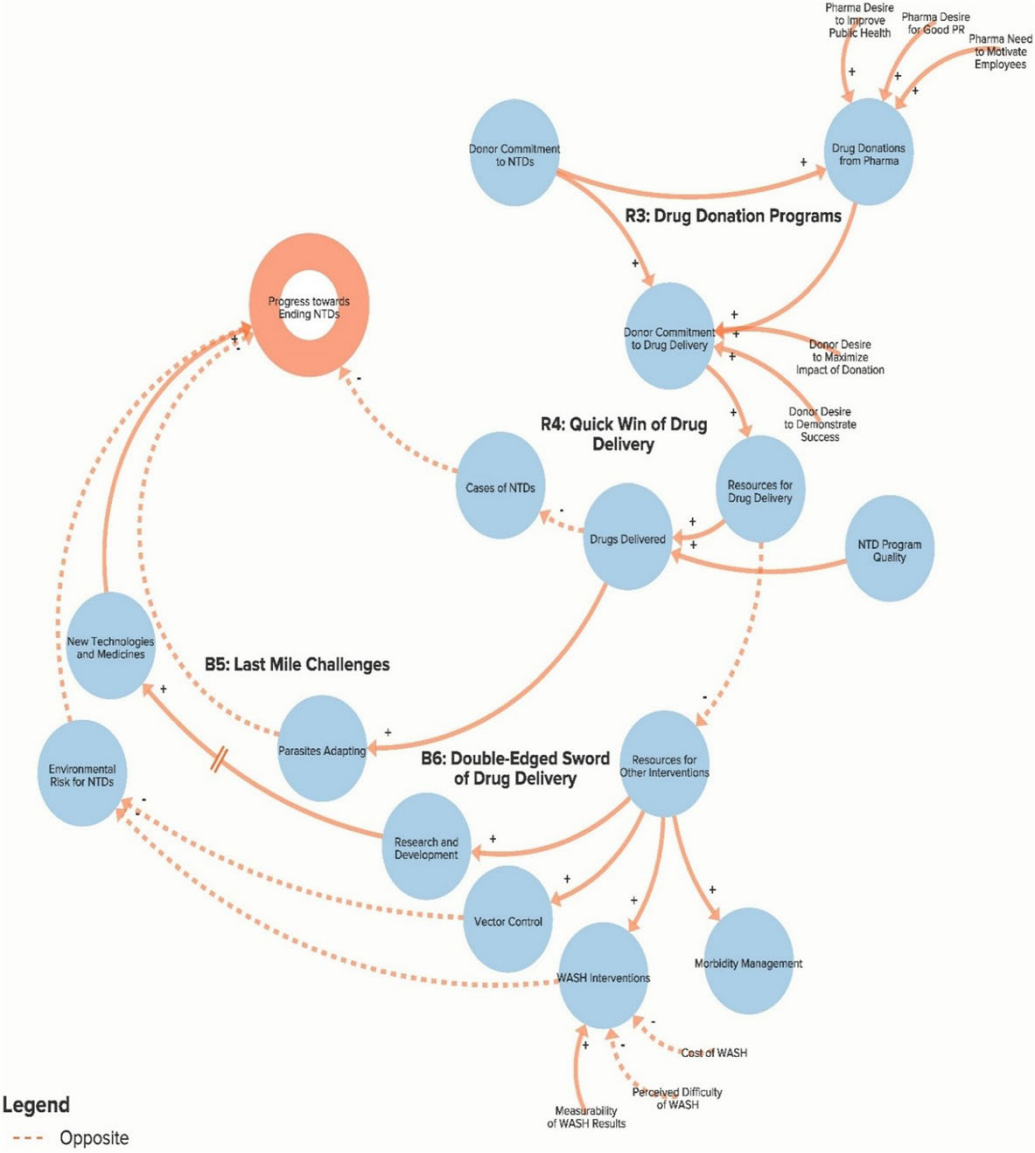
Sub-component 2: Focus of neglected tropical disease interventions

**Fig. 3:**
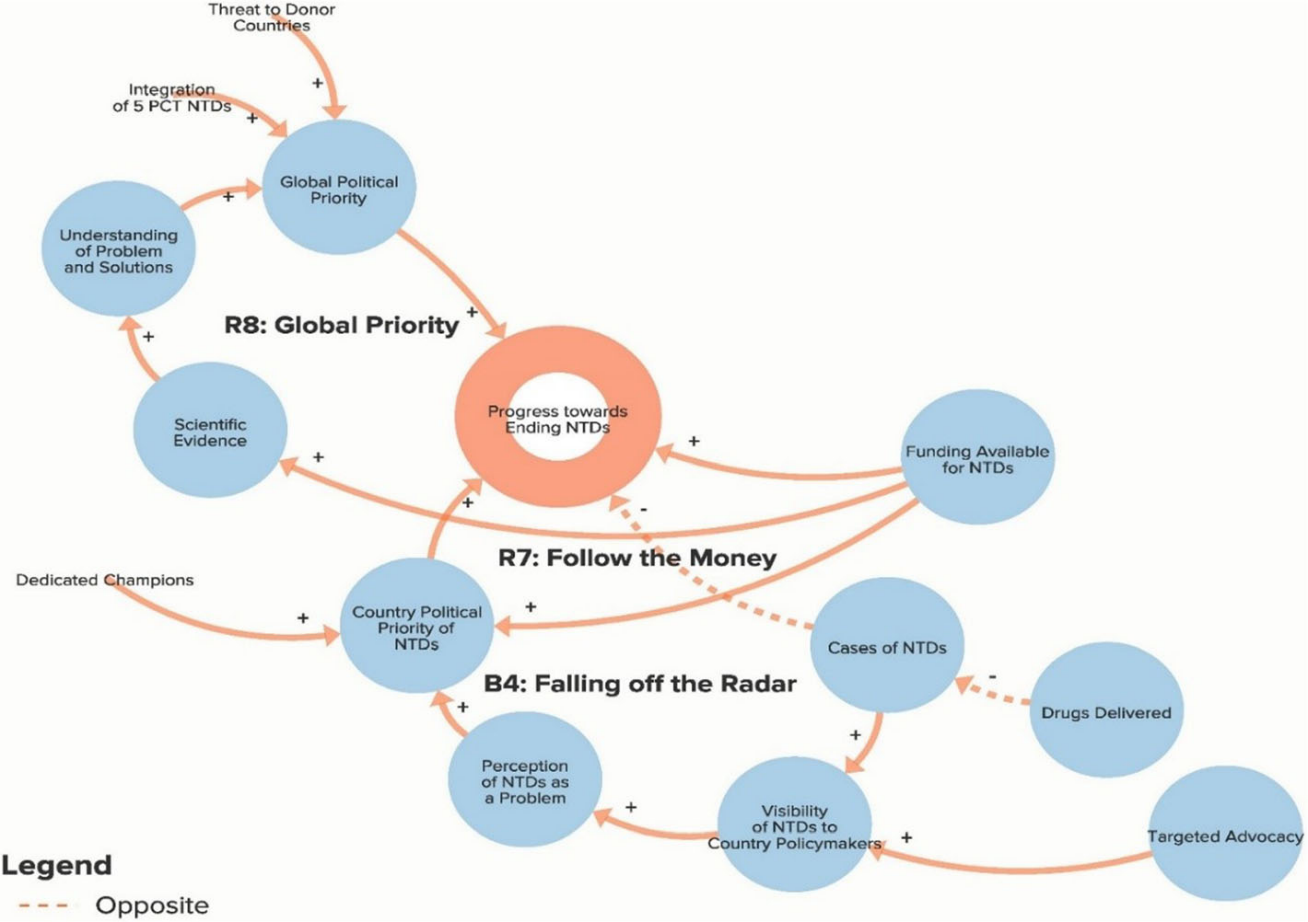
Sub-component 3: Political priority of neglected tropical diseases

**Fig. 4:**
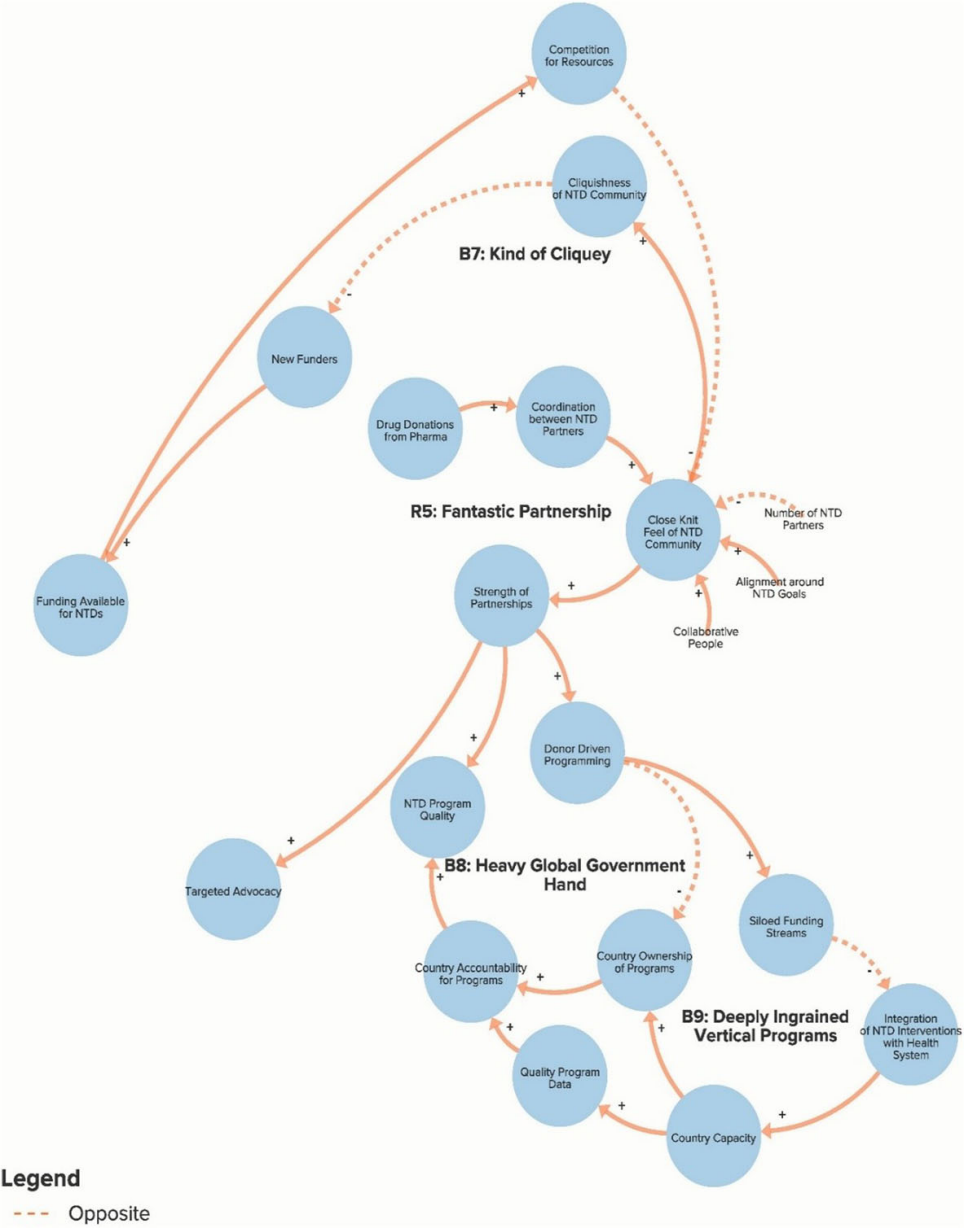
Sub-component 4: Global collaboration between neglected tropical disease partner organisations

**Fig. 5:**
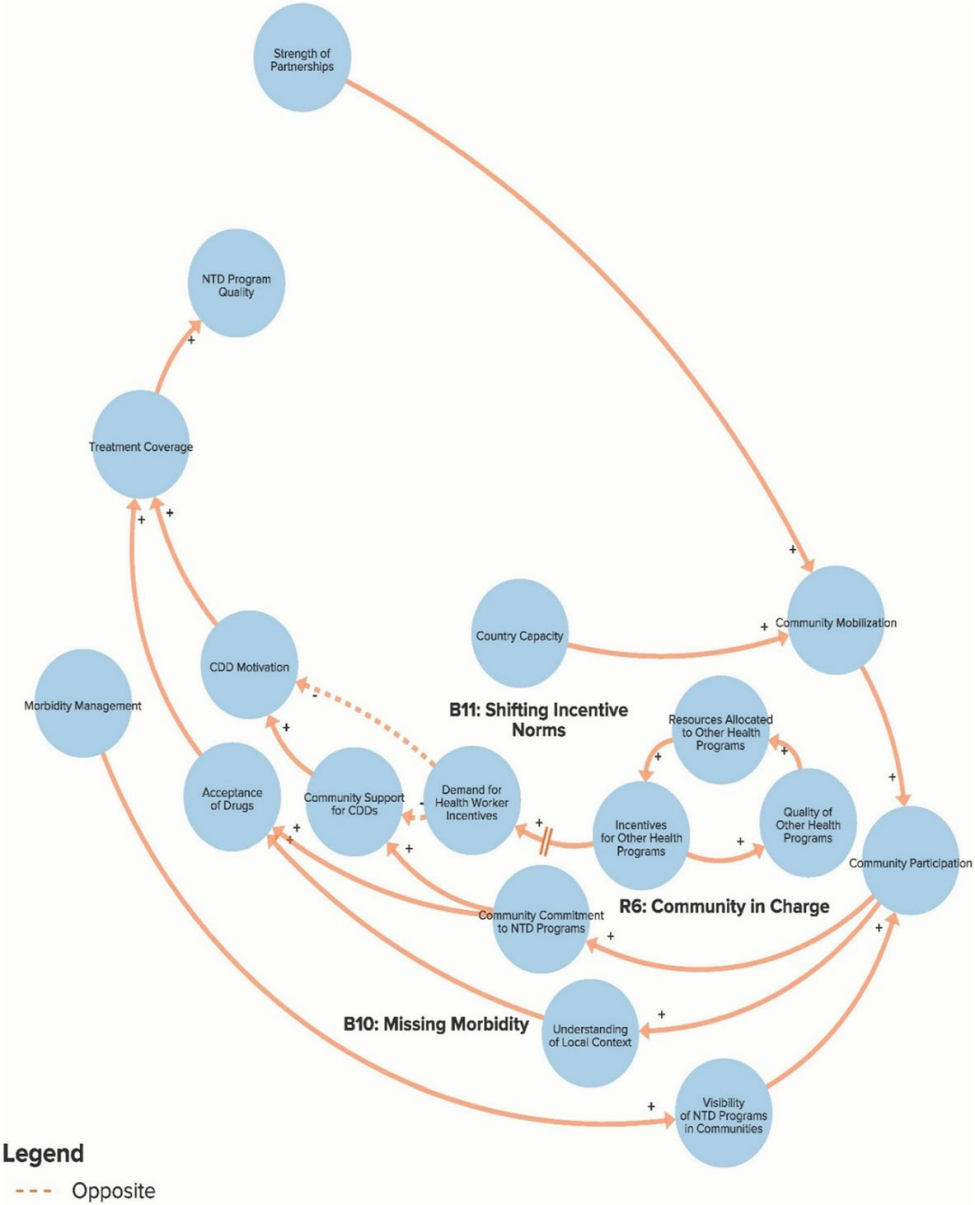
Sub-component 5: Community participation in neglected tropical disease programmes

**Fig. 6:**
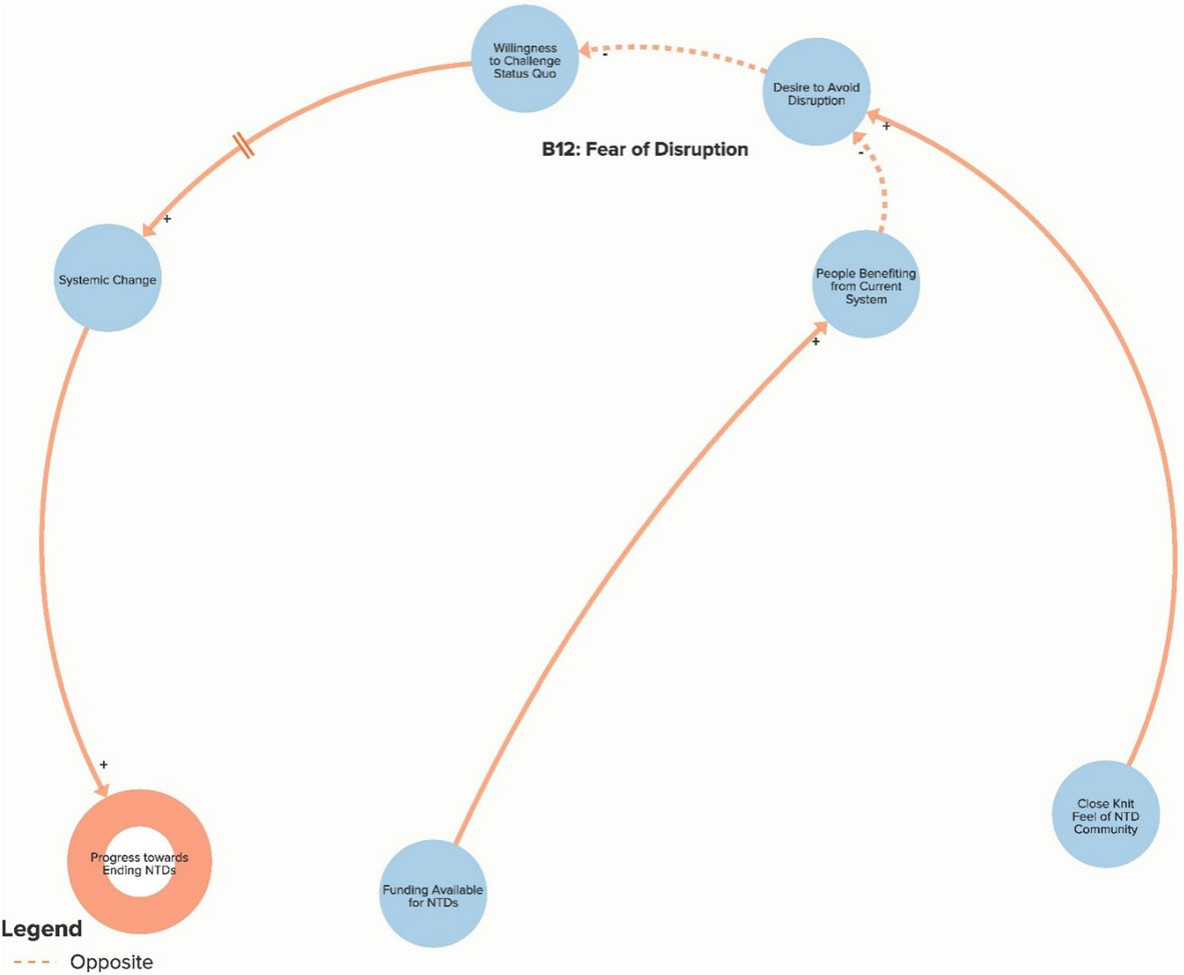
Sub-component 6: Introducing systemic change

**Fig. 7:**
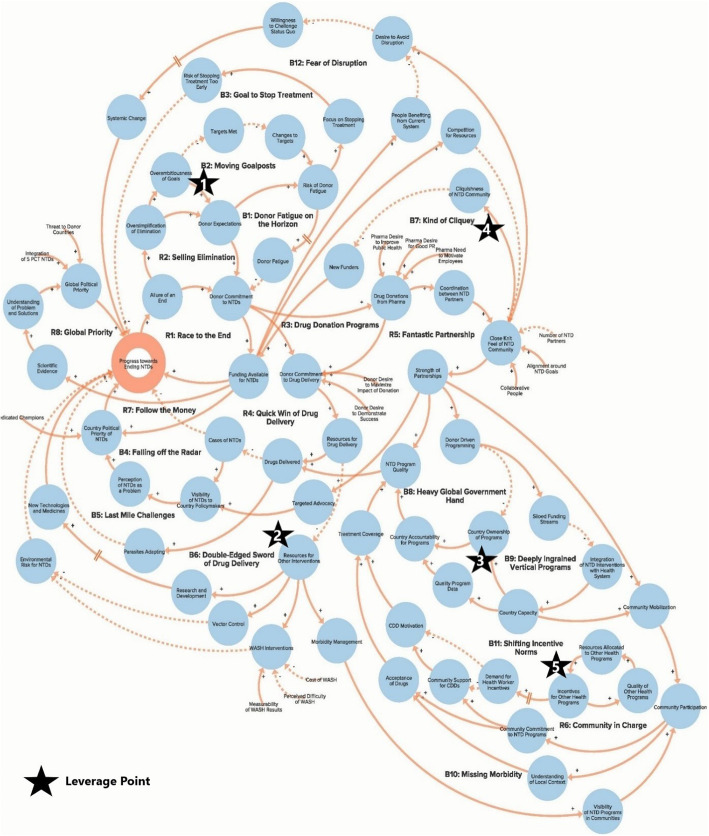
Complete qualitative model of the neglected tropical disease system (composed of six sub-components presented separately above)

### Global NTD advocacy

Figure 1 illustrates important forces related to global NTD advocacy. The two reinforcing loops, R1 and R2, highlight how the allure to donors of having specified and measurable NTD end goals contributes to increased donor commitment and funding as well as to an oversimplified advocacy message that increases donor expectations. While these factors have helped drive significant momentum for NTD programmes, high donor expectations and overambitious goals also increase the risk of donor fatigue (loops B1 and B2), which some stakeholders worry could eventually be a major barrier to progress as donors become disenchanted and withdraw funding before reaching NTD elimination goals. One key informant explained, “*It’s pretty clear that the situation could change and that these companies will have an appetite for these donations for only so long. And I don’t think anyone knows what that end point is, but I think people would rather not find out the hard way*” (Global NGO/Academic Stakeholder). At the same time, the risk of increased donor fatigue also encourages MDA programmes to focus on stopping treatment as soon as possible (loop B3) to demonstrate success to the donors. This could lead to a resurgence in disease prevalence if programmes are terminated prior to breaking disease transmission.

### Focus of NTD interventions

Figure 2 illustrates the key forces related to the various interventions used to control and eliminate NTDs. Reinforcing loops R3 and R4 outline the important role of the pharmaceutical companies’ drug donation programmes. As drug donations have increased over the past decade, donors have become more committed to drug delivery via MDA as the primary tool for addressing NTDs because they have a steady supply of drugs available and increased pressure to not waste them. While the massive influx of drugs and funding for drug delivery programmes has been successful in increasing the number of drugs delivered and reducing the overall cases of NTDs, an unintended consequence has been the neglect of other important complementary interventions (loop B6) such as water and sanitation infrastructure improvement, vector control, and research to develop new technologies. A global stakeholder described this problem, “*I think one of the major challenges for the NTD space is the prominence of the MDA effort. So the drug donation programmes are incredible. Full stop. They tend to be the focus of a lot of advocacy and promotional opportunities, which perhaps undermines opportunities to advocate for other aspects of disease intervention. I think the byproduct of that is that many funders very much focus just on the MDA component of any intervention. And it is quite hard to track funding for anything else*” (Global Multilateral/Bilateral Stakeholder). This represents a challenge because, as one Nigerian interview respondent told us, “*Everybody is focusing on MDA, MDA, MDA. Neglected Tropical Diseases will not depend on drugs alone. They will depend on water and sanitation. They will depend on morbidity management. They will depend on vector control. And they will depend on a number of things*” (Nigerian Multilateral/NGO/Academic Stakeholder). Other challenges (loop B5) that stem from the increasing number of donated drugs include the fear of increased potential for antibiotic resistance as parasites adapt to the existing drugs.

### Political priority of NTDs

Figure 3 illustrates the variables affecting the level of political priority that NTDs receive at the global and national levels. Reinforcing loop R7 shows the positive link between the level of international funding available for NTD programming and the prioritisation of NTDs at the country level. The existence of dedicated champions at the national level stood out as another key determinant of political priority. Looking at global political priority (loop R8), an increased understanding of the NTD burden and of viable solutions were factors reported to lead to increased prioritisation. The threat that donor countries perceive to their own citizens from NTDs was an additional factor believed to positively affect global political priority. The fact that this perceived threat is low offers one explanation for why global political priority is not as high as other diseases. As a stagnating force, balancing loop B4 highlights how a decreased NTD burden may lead to policy-makers seeing NTDs as less of a priority because the problems associated with the diseases become less visible and, thus, there is less urgency to address them. A global interviewee described the problem as follows: “*With any disease elimination programme, as disease prevalence goes down, it ceases to be the most pressing item on the public health agenda. So you always have a potential risk of you being in the wrong position on the carousel. So you reduce the prevalence, it falls off the radar, people stop complaining about it. It is deprioritised, and therefore you have prevalence reduced until such point as people say, ‘Oh, I thought we got rid of trachoma. We’ll have to go back and do that again’*” (Global Multilateral/Bilateral Stakeholder).

### Global collaboration between NTD partner organisations

Figure 4 illustrates important forces related to the active global network of NTD partner organisations. A common theme that emerged across many interviews was the strength and unity of the global NTD community, especially relative to networks focused on other global health issues. Reinforcing loop R5 suggests that this coordination can be partially explained by the need to collaborate to make use of the enormous amount of donated drugs, the alignment around a shared set of global goals, the small number of organisations dedicated to NTDs and the lack of competition for the relatively scarce pool of resources available. While interview respondents typically saw the strong partnerships as a positive force, the balancing loops in this picture highlight some potential counterintuitive unintended consequences that some respondents noted. Loop B7 shows how the close-knit feel of the community leads potential funders to perceive the NTD space as ‘cliquish’, which impedes new donors from getting involved. Loop B8 demonstrates how a united community of global partners may lead NTD programming to be more donor driven and thus inhibit country ownership and sustainable progress. One global interview respondent explained, “*Countries need to believe that these are their programmes. As long as they believe that they can get these things for free and that outside donors are doing these things and the medicine comes and the money comes, then you’re not going to see domestic funding. And my very strong opinion is that we’re in a time where we really need to put the countries in the driver’s seat in these programmes, such that they really start to own them. But right now I don’t think that they do and I think that WHO and the donors have set things up in a way that does not promote country ownership*” (Global NGO/Academic Stakeholder). Similarly, loop B9 suggests that siloed funding streams reduce programmatic capacity at the country level by preventing NTD programmes from becoming fully integrated into country health systems.

### Community participation in NTD programmes

Figure 5 illustrates variables related to participation at the community level in NTD programmes. Reinforcing loop R6 outlines how community mobilisation efforts are important determinants of community commitment and support for NTD programmes and the community drug distributors (CDDs) who deliver preventive chemotherapy drugs. Community participation also increases programme implementers’ understanding of the local context and leads to greater acceptance of drugs and, subsequently, higher treatment coverage. However, balancing loop B10 shows how focusing only on drug delivery and failing to address the morbidity of people with diseases reduces programme visibility in the community and leads to lower levels of community participation. Loop B11 highlights another impactful force that many Nigerian interview respondents saw as a major threat to NTD elimination efforts. (As depicted, B11 is not a true feedback loop because it is not shown to be affected by other factors in the model; however, it does represent an important set of variables that stagnate progress towards ending NTDs.) NTD drug delivery programmes typically rely on volunteer CDDs who sometimes receive small stipends. Donor-funded global health programmes for other diseases, such as polio and malaria, have increasingly been paying relatively large financial incentives to lay health workers to deliver programme services. Often, the NTD programme uses these same individuals as CDDs. While monetary incentives have increased the quality of these other programmes, this success has come at the expense of NTD programmes since community members are now less willing to deliver NTD services without receiving compensation. As one Nigerian respondent explained, “*That’s why an average health worker or community distributor does not want to do anything free because he knows or she knows that if he keys in to another programme he may be well compensated, better compensated. So it’s like a choice. You make* [a] *choice where you’ll be better remunerated*” (Nigerian State Government Stakeholder).

### Introducing systemic change

Finally, Fig. 6 helps explain the overall resistance to introducing systemic change into the global health community’s approach to addressing NTDs. Loop B12 emphasises how many individuals operating within the current system have a desire to avoid disrupting the status quo. This is partially attributable to the close-knit feel of the NTD community discussed earlier as well as to the benefits some people and organisations are receiving from the current system. In referencing changing global strategies to focus on elimination rather than merely control of NTDs, one global respondent explained, “*There have also been some institutions that were quite happy with the old control model which would focus just more on dealing with the morbidity reduction. … There’s a lot of … disagreement in the community about who wanted to move to the next agenda, who didn’t want to move to the next agenda, and getting clarity around the tools. I don’t think that was so much about the science and more about people wanting to protect the status quo*” (Global Multilateral/Bilateral Stakeholder). Another interviewee referred to large NGOs focused on NTDs, “*They’re all getting their funding from DFID* [United Kingdom Department for International Development) *and USAID* [United States Agency for International Development]*, and you wind up being seen as someone who’s sort of lobbying inconvenient truths at them that could potentially interfere with their funding. That’s the way they see it, so they tend to circle the wagons*” (Global NGO/Academic Stakeholder). As highlighted by our research, many people perceive a need for big changes (such as dedicating more resources to complementary interventions and to increasing country ownership of programmes) in order to achieve the ambitious NTD control and elimination goals. This overall desire to maintain the status quo acts a major barrier to making these necessary changes.

## Discussion

To our knowledge, this study is the first application of a systems thinking approach to the global health challenge of NTDs. Especially because this is a qualitative rather than a quantitative model, identifying leverage points for catalysing change depends, in part, on our subjective interpretation of the degree to which the stagnating power of the balancing loops outweigh the positive driving force of the reinforcing loops. After assessing instances in the model where altering the feedback loops or adjusting key variables would have a major positive impact on the fundamental structure of the system, we discussed our findings as a research team and identified five leverage points that we recommend global NTD stakeholders consider in order to accelerate progress towards global NTD elimination. Each of these points (highlighted in Fig. 7) is an area for intervention that can lead to change by weakening the key balancing feedback loops depicted in the model.

The first leverage point, to clarify the potential for and assess realistic progress towards NTD elimination, comes from the Global NTD advocacy sub-component (Fig. 1) of the model and would involve setting more plausible goals in order to address the resistance generated by loops B1 and B2 that increases the risk of donor fatigue. The second leverage point, to increase support for interventions besides drug delivery, comes from the Focus of NTD interventions sub-component (Fig. 2) and would focus on loop B6 by attempting to increase the priority given to WASH (water, sanitation and hygiene), vector control and other non-drug delivery interventions. The third leverage point, to reduce dependency on international donors, comes from the Global collaboration between NTD partner organisations sub-component (Fig. 4) and will depend on stakeholders’ ability to increase country capacity and ownership over NTD programmes in order to weaken loops B8 and B9. The fourth leverage point, to create a less insular culture within the global NTD community, comes from the same sub-component as the previous goal but addresses loop B7, in which the perception of a close-knit NTD community inhibits involvement from new actors. The fifth leverage point, to systemically address the issue of health worker incentives, comes from the Community participation in NTD programmes sub-component (Fig. 5), where failure to coordinate worker incentives across health programmes, as shown in loop B11, represents a major obstacle to programme coverage and quality. Although these are not necessarily completely novel ideas, our analysis clearly highlights their essential role in global efforts to control and eliminate NTDs. We also acknowledge that referring to these areas as leverage points may be a loose use of the term since they are likely to require more than merely small shifts to achieve the desired impact and since our model does not clearly articulate which specific interventions should be enacted. However, these areas for intervention nevertheless constitute an important starting point for inquiry and discussion about how to accelerate systemic change within the global NTD system.

A major strength of this study is its compilation of diverse perspectives to create a more extensive understanding of the complexity of the global NTD system. This range of perspectives illustrates systemic barriers preventing changes to the status quo since major disruptions have the potential to interfere with funding streams and partnerships that are currently beneficial to many stakeholders. We also found that the perspectives of global and local stakeholders were not always optimally aligned. For example, whereas global level respondents commonly described the need for countries to take ownership of NTD programmes, country level respondents sometimes perceived the actions of global organisations as undermining their ability to do so, especially due to funding earmarked only for certain activities or health service delivery programmes with competing incentive structures. These issues of donor dependency and health systems strengthening versus vertical approaches have been researched for decades in other areas of global health, but this study offers new insights from the systems perspective that highlight some negative consequences of top down, siloed approaches for NTDs [61–63].

The field of systems thinking faces a gap between theoretical discussions about and real-world progress towards positive systemic change. Our research offers a path forward by illustrating how the academic concepts and tools of systems thinking can be used in an actionable way that informs the strategy of change agents working on large-scale systems change efforts to solve society’s most complex challenges. It also supports the argument made by others that a systems perspective is needed in the field of global health [7, 27, 34].

This study is not without its limitations. The variables and relationships represented in this model highlight a sample of issues that stakeholders reported as key factors impeding progress on NTDs and representing opportunities for systems change, but they do not necessarily encompass a comprehensive view of the entire NTD system. Although the goal of the model was to include the most relevant variables and linkages that were explicitly identified in the data, selecting key themes to highlight and determining the number of intermediary variables to include in a causal link between two key variables is somewhat subjective. Thus, there is still a risk that researcher bias has impacted the model.

Systems theory suggests that the perspectives included in our results are important because at least some stakeholders see them as barriers, but the analysis conducted here does not claim to determine the degree to which they actually represent significant challenges to progress. Thus, each of the issues discussed in the results section merits further consideration and research. Additionally, although interviews were conducted with a large number of stakeholders, some perspectives remain unrepresented. For example, interviews did not include stakeholders working on NTD research and development, individuals involved with other health and development issues, or community members living in NTD endemic areas. Future research should address these gaps. While there were opportunities for some stakeholders to provide feedback on the model, a more participatory and iterative model building process with a broader range of stakeholders would increase the validity of the results. Finally, each country and community faces unique challenges that are obviously not covered by focusing at the sub-global level on one example country, as we did in this analysis with Nigeria. While the global level interviews addressed cross-national issues, and while it is likely that the experiences of Nigerian NTD programmes are in some ways similar to those in other countries, we cannot necessarily generalise these findings from Nigeria to other places.

## Conclusion

This study provides an example of academically rigorous qualitative research combined with a practical conceptual framework for applied systems thinking in order to identify levers for large-scale change. It also recommends five specific leverage points for NTD systems change, namely (1) clarify the potential for and assess realistic progress towards NTD elimination, (2) increase support for interventions besides drug delivery, (3) reduce dependency on international donors, (4) create a less insular culture within the global NTD community, and (5) systemically address the issue of health worker incentives. The specific findings for NTDs raise a number of questions that have not been addressed, at least in part, because it is easier to continue focusing on ‘quick win’ solutions. Systems change for NTDs, as with change in all complex contexts, can only be achieved through continual collaboration that generates joint learning and innovation.

There is a dearth of evidence in the systems change literature of successful examples of prospective systems change approaches applied to complex social problems. A next step for the field of systems thinking, and one for which documented examples from both the academic and practice sectors are needed, is to conduct rigorous assessments of the process and outcomes of collective efforts to implement systems thinking-generated recommendations [19, 20]. Such research should attempt to track the ways in which systems actually change as well as the ways in which stakeholders’ mental models and perspectives change throughout the process. While large scale social change will almost always be a challenging and lengthy pursuit, more robust evidence around successful and failed attempts to apply systems thinking approaches would be a valuable contribution to the field. 

## Data Availability

The datasets generated and/or analysed during the current study are available from the corresponding author on reasonable request.

## Abbreviations

CDD: Community Drug Distributor
MDA: Mass Drug Administration
NGO: non-governmental organisation
NTD: Neglected Tropical Disease
